# Detecting the Deformation Behavior of Bimodal Ti-6Al-4V Using a Digital Image Correlation Technique

**DOI:** 10.3390/ma15217504

**Published:** 2022-10-26

**Authors:** Mei-Yue Li, Bin Zhang, Zhu-Man Song, Xue-Mei Luo, Guang-Ping Zhang

**Affiliations:** 1 Shenyang National Laboratory for Materials Science, Institute of Metal Research, Chinese Academy of Sciences, 72 Wenhua Road, Shenyang 110016, China; 2 School of Materials Science and Engineering, University of Science and Technology of China, Shenyang 110016, China; 3 Key Laboratory for Anisotropy and Texture of Materials (Ministry of Education), School of Materials Science and Engineering, Northeastern University, 3-11 Wenhua Road, Shenyang 110819, China

**Keywords:** Ti, alloy, metal, ductility, stress/strain relationship

## Abstract

The evolution of a local strain of the Ti-6Al-4V alloy subjected to tensile loading was investigated in situ by using the digital image correlation technique. The results show that some local strain concentration areas have already appeared in the elastic deformation stage, which then connected and became concentrated in the gauge region when the specimen yielded. The strain compatibility of grains in the macroscopic region is kept constant. The deformation process is further divided into six parts based on the development of the maximum strain gradient, and the strain compatibility of each stage of the alloy is summarized and analyzed. The quasi-in situ experiment reveals that the primary *α*(*α_p_*) grains undertake the main deformation at the micro-scale.

## 1. Introduction

Titanium alloys are widely applied in the aerospace industry for their high specific strength, corrosion resistance, and good fatigue performance [1]. One of the most popular Ti alloys for aerospace applications is Ti-6Al-4V (TC4), which is a typical *α* + *β* Ti alloy. The metal material with a hexagonal close-packed (HCP) structure shows obvious deformation inhomogeneity during tensile loading due to the anisotropy of the internal structure of the material, which leads to the inhomogeneous distribution of the strain [2,3,4]. Hence, it is important to understand the local deformation behavior of the material during tensile loading. Luster and Morris [5] proposed the concept of a geometric compatibility factor to determine the precise spatial relationship among grains. They found that the orientation relationship affected the deformation process. After that, a large number of studies have been conducted on strain compatibility in the deformation process of metal materials, among which in situ observation and characterization are considered to be favorable experimental means [6,7]. For example, the digital image correlation (DIC) technique is one of the most important non-contacting methods to measure full-field displacement, which has been widely applied to composite structures [7,8], construction material applications [9], and biomedical materials [10]. Dunne et al. [11] used the DIC technique to measure the local deformation of single-crystal nickel superalloy at room temperature. They found that the geometric necessary dislocations (GNDs) observed in the elastic deformation stage were related to the plastic strain gradient. Xia et al. [12] carried out an in situ tensile test on an AZ31 magnesium alloy in a scanning electron microscope and found that strain concentration appeared in the early stage of tensile deformation. Similarly, Zhu et.al [13,14] also found that the heterostructure can induce dispersive shear bands over the whole gauge section using the DIC technique, and the shear bands nucleate at the early strain stages. Some studies have described strain compatibility in the tensile process of titanium and titanium alloys [15,16]. Ye et al. [16] show that the twins formed at the common boundary of two grains in pure titanium have good strain compatibility, while the twins formed at the triple point show poor strain compatibility. Li et al. [15] proposed that the yield strength and stress compatibility coefficient decreased with the increase in the *α* phase content in the TC4 titanium alloy with no texture at high temperatures. Nonetheless, how the strain localization evolves in the whole tensile process of metals has not been characterized well. 

In this study, the evolution of the local strain of a bimodal TC4 specimen was evaluated under in situ uniaxial tension by using the DIC technique. The full-field strain nephograms were obtained to establish the relationship between local strain and macro tensile deformation, from which the local deformation mechanism of the bimodal Ti-6Al-4V specimen was discussed.

## 2. Materials and Methods

### 2.1. Material and Specimen

**Figure 1:**
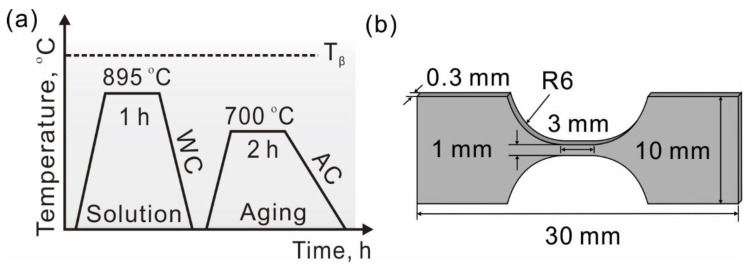
Schematic illustrations of (**a**) the process of heat treatment and (**b**) tensile specimen.

The material investigated here is a commercial TC4 alloy (Nominal composition: Al: 6 wt%, V: 4 wt% with the rest being Ti). The alloy was subjected to solution treatment at 895 °C for 1 h and subsequent water cooling. Then, the alloy was aged at 700 °C for 2 h followed by air cooling, as shown in Figure 1a. Microstructures of the alloy were characterized by scanning electron microscopy (SEM, LEO Supra 35). The alloy is a bimodal structure consisting of equiaxed primary *α* (*α_p_*) grains and lamellar secondary *α* (*α_s_*) colonies. The volume fraction of the *α_p_* phase in the alloy is about 76%.

### 2.2. Tensile Testing and Characterization Method

Small tensile specimens with gauge dimensions of 3 mm long and 1 mm wide were prepared using an electric spark discharge wire cutter (SKD3); the schematic diagram of the specimen is shown in Figure 1b. To achieve this measurement, all the specimens were mechanically ground using SiC papers from 400 # to 3000 # and then polished mechanically using colloidal silica suspension. Finally, the specimens were chemically etched in a solution of methyl alcohol (59.4%), n-butyl alcohol (34.6%), and perchloric acid (6%) at 20 V/253 K to reduce the residual stress and obtain a smooth surface. The final thickness of the specimens was about 0.3 mm.

In situ tensile tests were conducted in an Instron 5848 tester at a strain rate of 5 × 10^−4^ s^−1^ at room temperature. The test was repeated on three specimens at least to ensure data reproducibility. In order to monitor the transient strain at a miniature specimen surface with tensile loading, the DIC technique was set up for its high resolution and no contact with specimen surfaces. DIC measurement is based on computing displacements and displacement gradients of material points between a reference (undeformed) image and a subsequent (deformed) image from synthetic speckle images which requires good contrast and very fine patterning [17]. The main part of the DIC system is a camera which consists of a Prosilica GT1600C camera (1620 × 1220, 25 fps) and a Work Power lens (WP-5MO.18X110, *f*_0_ = 110 mm). Before the tension test, the specimen surfaces were sprayed with black dots to make sure the camera can track the position. The digital speckle images of the specimen gauge section were collected by the camera at every 1 s. Upon tensile loading, the camera began to record the images. Finally, the GOM Correlate software was used to calculate the local strains by the change in the speckle positions according to the images at different loading times.

The surface morphology and crystallographic orientations of the fracture specimens were characterized by using SEM with an electron backscattering diffraction (EBSD) detector.

### 2.3. Quasi-In Situ Tensile Testing Method

The specimen of the quasi-in situ experiment is the same as that of the tensile specimen. The surface of the specimen was carefully ground and electropolished for the EBSD observation. The observation site after each stop of the dynamic deformation was unchanged. Then, a 40 μm × 40 μm area for observation was selected at the center of the gauge section.

To investigate the microstructural evolution, a quasi-in situ characterization method was established [18]. The principal procedure is as follows, (1) The initial microstructure of the marked area of the specimen was characterized by EBSD. (2) The specimen was subjected to a defined strain at a strain rate of 5 × 10^−4^ s^−1^ and then unloaded the force to zero at the same strain rate. (3) The microstructure of the marked area for the first deformation cycle was characterized by SEM and EBSD. (4) A higher strain was applied to the specimen at the same strain rate, and (5) The microstructure for the second deformation cycle was observed. Then, the “dynamic loading with a strain increment—microstructure recording at the same area” process was repeated on the same specimen. Following these steps, the microstructure evolution of a certain area in a specimen subjected to dynamic tension can be recorded.

After each dynamic loading break, the specimen was only cleaned with acetone to reserve the surface deformation morphology, which was examined by SEM, while the crystallographic orientations of each grain were gathered using EBSD. The crystallographic orientation data and the local misorientation data were analyzed using the HKL channel 5 software. For kernel average misorientation (KAM) mapping, each EBSD scan was performed with a step size of at least 50 nm to ensure enough spatial resolution.

## 3. Results

### 3.1. Heat Treatment 

**Figure 2:**
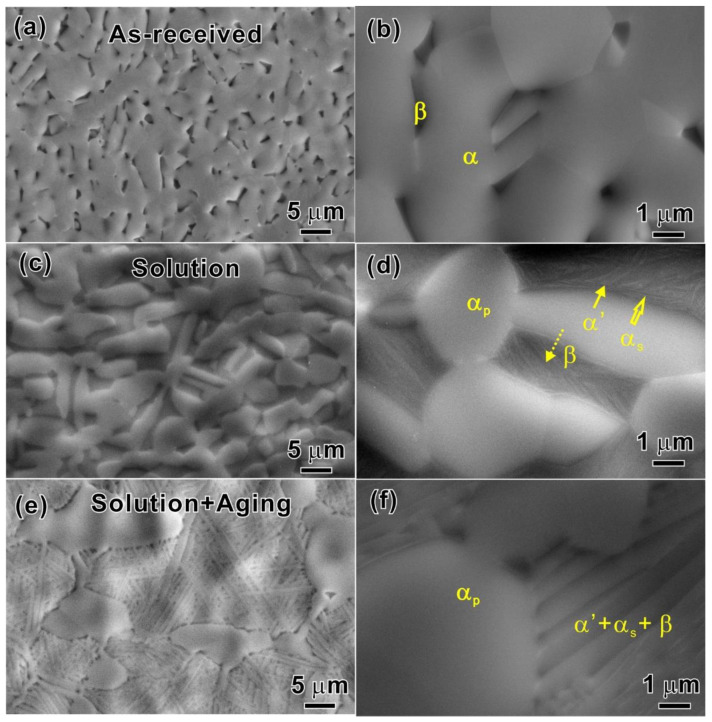
(**a**) As-received microstructure. (**c**) nearly equiaxed and elongated *α_p_* microstructure with the formed *α′* martensite, *α_s_* and retained *β* phases after ST at 895 °C followed by water cooling. (**e**) bimodal microstructure after solution and aging treatment. (**b**,**d**,**f**) the magnified observations of (**a**–**c**), respectively.

SEM micrographs of as-received and heat-treated specimens are shown in Figure 2. The as-received microstructure shown in Figure 2a has a typical dual-phase microstructure (*α* + *β*); the magnified observation is shown in Figure 2b. On solution treatment (ST) at 895 °C followed by water cooling, a nearly equiaxed and elongated microstructure with predominant *α_p_* grains is received, as shown in Figure 2c. A much smaller fraction of *α′* (HCP-martensite shown by the solid arrow) formed at a high cooling rate and *α_s_* (the very fine needle-shape shown by the hollow arrow) in the *β* phase. The residual *β* phase is shown by a dotted arrow, as shown in Figure 2d. After the ST, the alloy was aged at 700 °C for 2 h, followed by air cooling. The bimodal microstructure consisting of 76% equiaxed *α_p_* phase and basketweave microstructure with the coarse lamellar *α_s_*, ultra-thin retained *β* and *α′* martensite phases were formed, as shown in Figure 2e and the magnified observation in Figure 2f.

### 3.2. Deformation Process 

**Figure 3:**
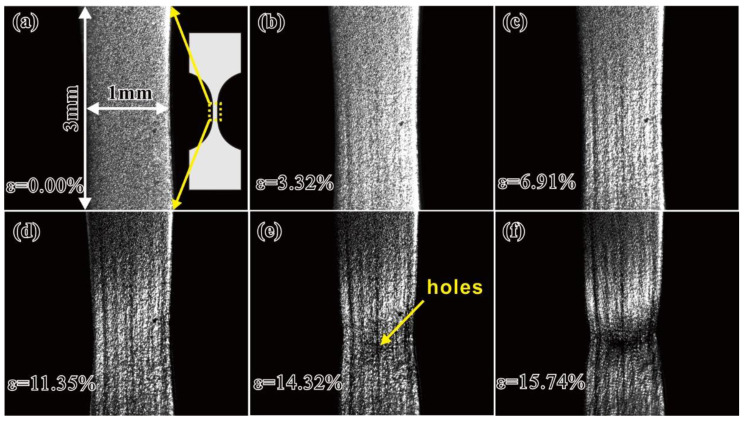
Deformation evolution at the surface of the specimen subjected to different strains from (**a**–**f**) during tensile loading.

Figure 3 shows in situ optical images of the specimen surface at different tensile strains (*ε*). An inset in Figure 3a shows a schematic diagram of the tensile specimen. As the applied tensile strain *ε* = 3.32%, some deformation bands parallel to the loading direction appeared on the specimen surface, as shown in Figure 3b. As *ε* = 6.91%, necking occurred (Figure 3c). As *ε* = 11.35%, the specimen surface became more uneven, as shown in Figure 3d. With further increases in *ε* to about 14.32%, the macro holes appeared at the specimen surface, as indicated by an arrow in Figure 3e. Finally, the specimen broke at *ε* ≈ 16% (Figure 3f), and serious distortion bands formed at the specimen surface.

### 3.3. Evolution of Local Strain under Tensile Loading

**Figure 4:**
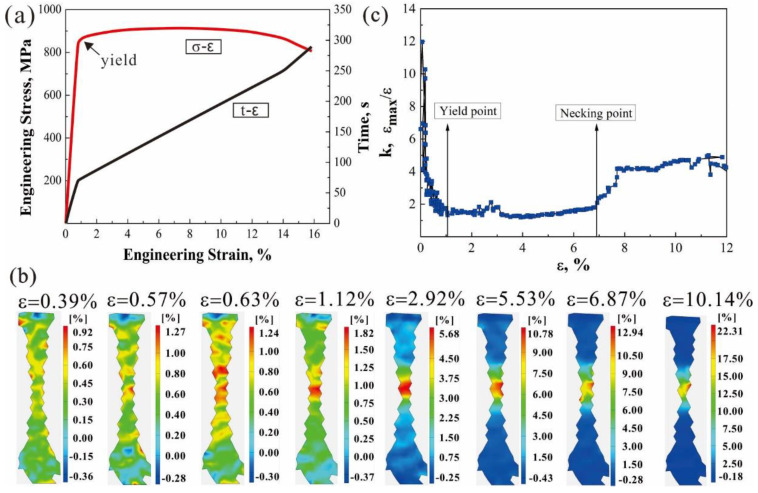
Tensile property of the TC4 alloy: (**a**) engineering stress–strain curve, (**b**) the full-field strain nephograms of the gauge section at different tensile strains, and (**c**) the strain concentration factor-strain curve.

Figure 4a presents an engineering stress–strain curve and loading time (*t*) vs. the corresponding strain curve of the specimen. The mean strain (*ε*) characterized by an average principal strain of the whole gauge section is measured by DIC. The specimen yielded at *ε* = 1.12%. Figure 4b shows the full-field strain nephograms of the gauge section at different tensile strains. In the elastic deformation stage (0 < *ε* < 1.12%), there are about 5–8 discrete strain concentration regions on the specimen surface, as shown in the red zones in the first three nephograms in Figure 4b, which indicates that the local plastic deformation has occurred in the elastic stage. Lunt et al. [19] also found that the local plastic deformation of a TC4 alloy was formed in the early stage of plastic deformation. When the specimen yielded, these discrete strain concentration regions coalesced and concentrated in the gauge section, as shown in the fourth nephogram in Figure 4b. This phenomenon is also observed in a magnesium alloy during in situ tensile loading [12,20].

In order to characterize the degree of strain concentration in the local area of the specimen, the strain concentration factor (*k*) [21,22,23] is introduced by

(1) $$k=\frac{\varepsilon_{max}}{\varepsilon }$$

where *ε_max_* is the maximum principal strain; Figure 4c shows the variation of the calculated strain concentration factor *k* with the tensile strain. When 1.12% < *ε* < 6.87%, the specimen is subjected to uniform plastic deformation, and *k* is close to 1 and remains unchanged, i.e., *ε_max_* ≈ *ε*. This indicates that the width of the strain concentration area is approximately equal to the length of the gauge section. The *k* value increases abruptly at about *ε* = 6.87%, indicating that the strain is further concentrated in the center of the gauge section, while the maximum strain in the strain concentration area reaches 12.94%, and then the specimen begins to neck and gradually fractures. 

**Figure 5:**
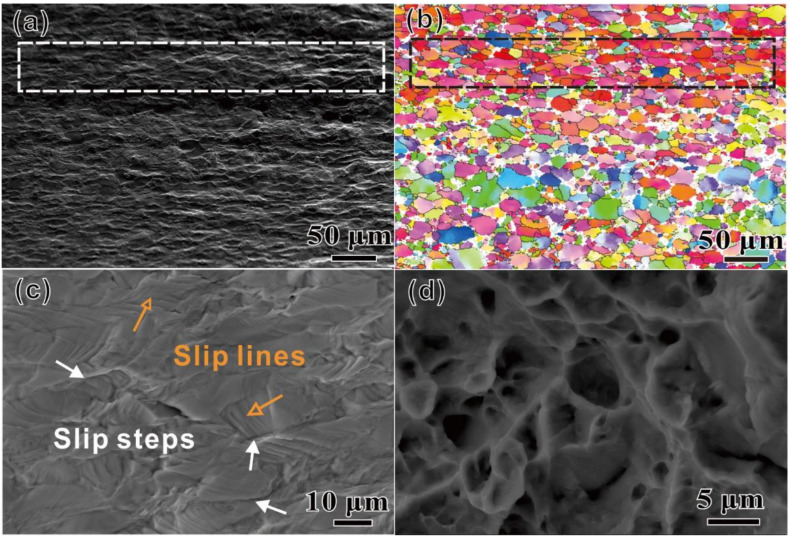
Surface characterization of the fractured specimens. (**a**) SEM and (**b**) EBSD images with macro zone after fracture. (**c**) SEM observation images of the surface morphology of the tensile specimens, and (**d**) the fracture surface.

Figure 5a,b show an SEM image of the deformation bands at the specimen surface after fracture and the corresponding EBSD grain orientation map, respectively. Crystallographic orientations in the deformation band are quite similar, as shown in the black dotted bordered rectangle. The region consisting of several adjacent grains with similar crystallographic orientation is called the “macrozone” or microtexture, which is a set of neighboring individual grains with the similar crystallographic orientation that can potentially act as structural unit regions and can be deformed as an independent unit during tensile loading [19]. This difference in crystallographic orientations in different macro zones results in highly uneven deformation [24,25]. Thus, the occurrence of the fluctuation of deformation bands occurs and the increase in the corresponding surface roughness may result in the initiation of microcracks, as indicated by the white dotted bordered rectangle in Figure 5a. Figure 5c shows a close SEM observation of the deformation morphology at the specimen surface. Many slip lines and slip steps appeared in the *α_p_* grains, as indicated by the hollow arrows and solid arrows, respectively, indicating that the slip is the main deformation mode for the specimen. In addition, the fracture surface shows obvious dimple morphology, as shown in Figure 5d, which is the typical feature of ductile fracture.

## 4. Discussion

### 4.1. Evolution of Deformation Calculated from DIC Data during Tensile Test

**Figure 6:**
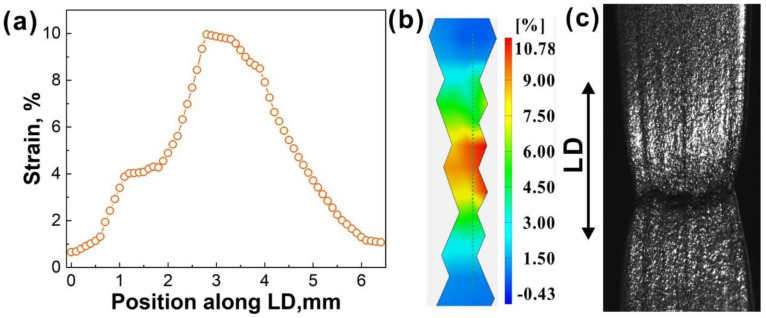
Deformation localization: (**a**) the strain along the loading direction, (**b**) strain nephogram, and (**c**) specimen after fracture.

The local strain is an important factor affecting the tensile properties. The tensile failure would occur once the plastic deformation is highly localized. By selecting a series of points evenly along the loading direction (LD) at the center of the gauge section on the specimen surface at the *ε* = 6.52% which is close to necking, the local strain along the LD is obtained, as shown in Figure 6a. Figure 6b is the strain nephogram showing the positions selected for the points. This indicates that the local necking occurring at the center of the specimen causes a sudden initiation of a crack which will propagate rapidly to failure. Figure 6c shows that the final failure path is along the transverse direction.

The inhomogeneous development of the local strain within grains and the deformation incompatibility among grains may need the accommodation of GNDs [26]. To estimate the GNDs caused by the plastic strain gradient in the specimen, the strain gradient (*η*) is defined as a ratio of the principal strain difference (∆*ε_p_*) to the distance between two adjacent points (∆*x*),

(2) $$\eta =\frac{\Delta \varepsilon_{p}}{\Delta x}$$

The density of GNDs (*ρ_G_*) can be related to the plastic strain gradient by introducing the Nye factor [27], i.e.,

(3) $$\rho_{G}=\bar{r}\eta ∕b$$

where $\bar{\gamma }$ is used to measure the GND density generated by the macroscopic plastic strain gradient [28], and *b* is the Burgers vector.

**Figure 7:**
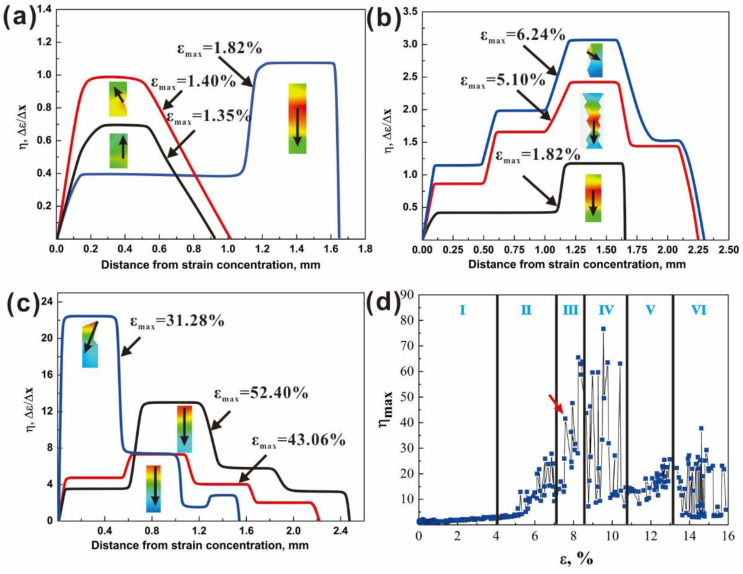
Relationship between the strain gradient and the distance from the strain concentration at different applied strains in the (**a**) elastic, (**b**) work hardening, and (**c**) instability stages, respectively. (**d**) The maximum strain gradient–strain curve.

To study the evolution of the GND density along the tensile direction, we calculate the strain gradient at different deformation stages. Figure 7a–c show the change in the strain gradients with increasing distance to the strain concentration area at several typical loading times corresponding to the elastic, work hardening, and deformation instability stages. The curves with different maximum principal strain *ε_max_* values in the strain concentration area at these stages are presented in Figure 7a–c, respectively, and the insets show the nephograms of the gauge region, in which we create a series of points in the direction of the fastest strain decline along the arrow until the strain is close to zero. Generally, the maximum strain gradient *η_max_* increases with the increase in *ε_max_*. Comparing the elastic deformation, the work hardening and the deformation instability stages are shown in Figure 7a–c, respectively, after yielding (1.82% ≤ *ε_max_* < 52.40%); the distance from *ε_max_* to *ε* = 0 at the work hardening stage is the largest as the dislocation slips are extensively activated in more grains to accommodate plastic strain [29]. In addition, there is a periodic process before *η* up to *η_max_*; that is, the strain gradient has a sustained increase at the first 25 grains (the average grain size is 5 μm) and then remains unchanged subsequently with a range of 75 grains, which indicates that the strain accommodation ability of these 75 grains keeps constant, meaning that they belong to the same macro zone. At the same time, the scale of the strain accommodation region is about three times that of the strain concentration region, which is only applicable to the particular geometry of the specimens in this study. As shown in Equation (3), the strain gradient is proportional to the GND density, so the change in the dislocation density can be measured by the change in the strain gradient. Dislocations in the strain concentration region produce a high local stress concentration, which stimulates adjacent grains to generate dislocations [30]. This stress concentration cannot be alleviated until the dislocations can supplement the strain gradient caused by the strain concentration.

Because the strain gradients at different displacements at the same strain are different, to simplify the analysis, we take the maximum strain gradient (*η_max_*) to represent the strain accommodation ability of the specimen at this strain. If the strain gradient is large, the required strain accommodation degree is large. Figure 7d shows the relationship between *η_max_* and the strain *ε*. According to the characteristics of the curve, the whole tensile process is divided into six parts:

(1) Region I is the elastic deformation stage (0 < *ε* < 1.12%). 

(2) Region II is the work-hardening stage (1.12% < *ε* < 5.33%). The *η_max_* increases uniformly with increasing the strain, indicating that dislocation slip is the main way to coordinate the strain, and the dislocation density increases with increasing the strain.

(3) Region III is the necking stage (5.33% ≤ *ε* < 6.87%). The *η_max_* reaches the first peak at *ε* = 6.87%, as indicated by the red arrow. This is attributed to an increase in the strain accommodation degree caused by the accumulation of dislocations at the grain boundary [31].

(4) Region IV is the first deformation instability stage (6.87% ≤ *ε* < 10.45%). When further increasing the strain, *η_max_* suddenly rises to the maximum value and then drops to a low point. This is because the extensive grain boundary sliding of TC4 may occur at room temperature [32], which is evidenced by local regions indicated by the white arrows in Figure 5c.

(5) Region V is the second deformation instability stage (10.45% ≤ *ε* < 13.97%). Further deformation causes the local strain to rise again, so the *η_max_* begins to increase again.

(6) Region VI is the third stage of deformation instability. The crack develops rapidly. Some grains no longer participate in the strain accommodation. Therefore, the strain compatibility degree fluctuates severely.

The above quantitative characterization clearly reveals that the strain compatibility and deformation instability features of the TC4 alloy during tensile loading are mainly related to dislocation and grain boundary activities in the *α* grains. The rapid increase in the maximum strain gradient in the strain hardening stage and subsequent necking implies that plastic deformation needs more dislocation slips and even grain boundary sliding. The strain gradient appears as an obvious inflection point before the *η* keeps constant, indicating a significant plastic deformation incompatibility between the two macro zones on both sides of the inflection point [33]. At the same time, the dislocations undergo a periodic process of activation and transmission. The activated dislocations need to slip a triple length of the strain concentration region to alleviate this strain concentration, then accumulate in the next macro zone to create another concentration area until the strain becomes zero. Although there have been a lot of investigations on the tensile deformation behaviors of Ti alloys with different microstructures, here, the present findings derived from the in situ DIC technique may provide a deep understanding of how plastic deformation and its compatibility develop locally at a macro scale.

### 4.2. Evolution of Deformation Observed Directly by Quasi-In Situ Experiment

In Section 4.1, the evolution process of deformation is inferred indirectly from the dislocation density calculated by DIC. However, this method cannot reveal how the bimodal microstructure influences the deformation process during tensile loading. To reflect the influence of the bimodal characteristic of grain size on the evolution of dislocation density and the deformation process more intuitively, a quasi-in situ experiment was conducted.

**Figure 8:**
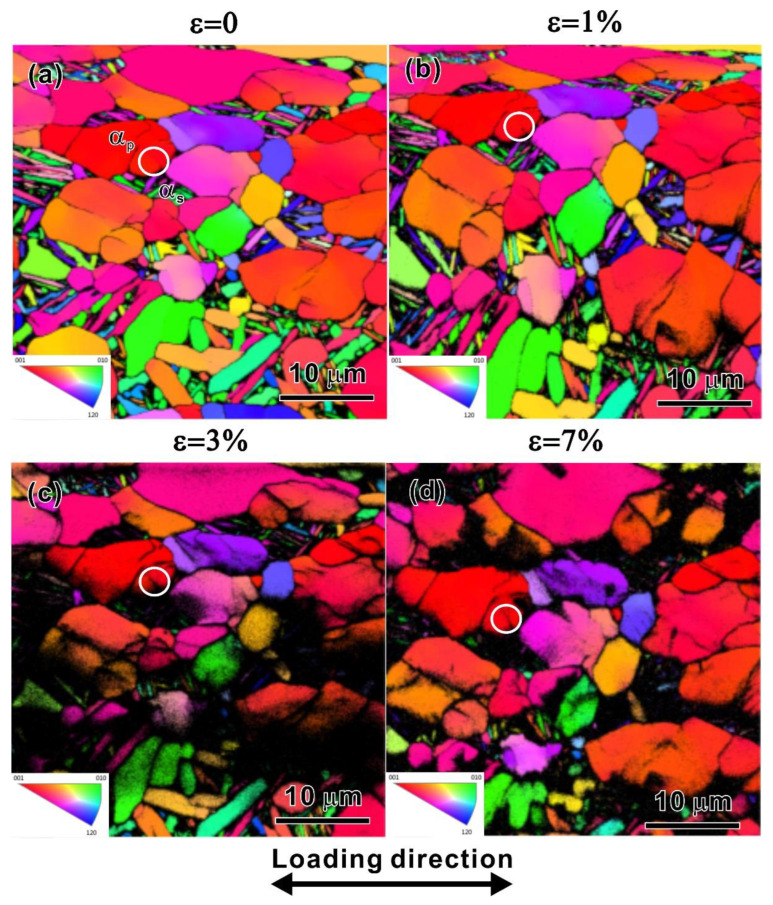
IPF maps of the observation area with strains of (**a**) 0%; (**b**) 1%; (**c**) 3%; and (**d**) 7%, respectively, in a quasi-in situ tensile test.

**Figure 9:**
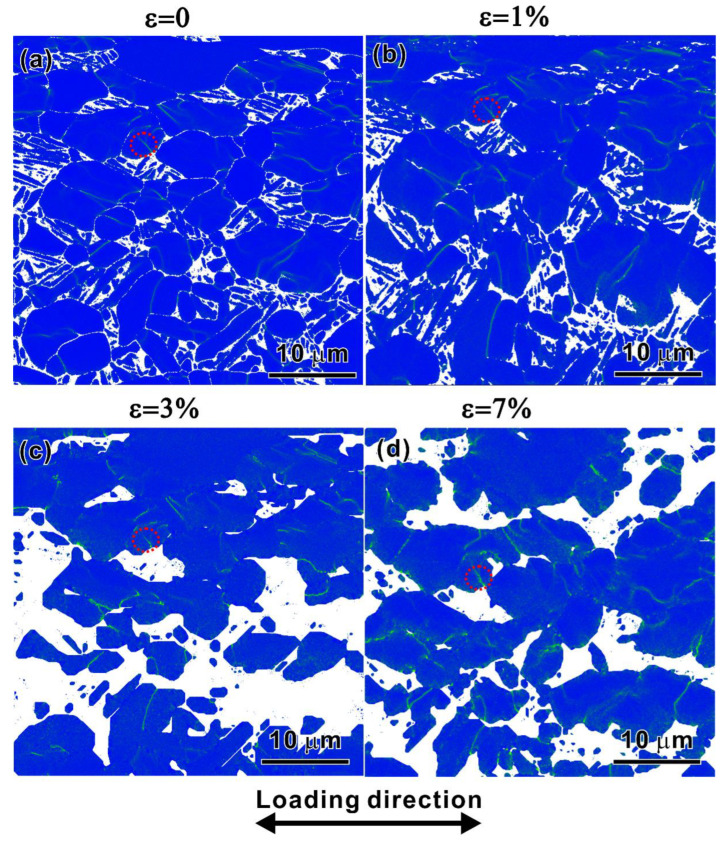
GND density mapping based on the EBSD data with strains of (**a**) 0%; (**b**) 1%; (**c**) 3%; and (**d**) 7%, respectively.

**Figure 10:**
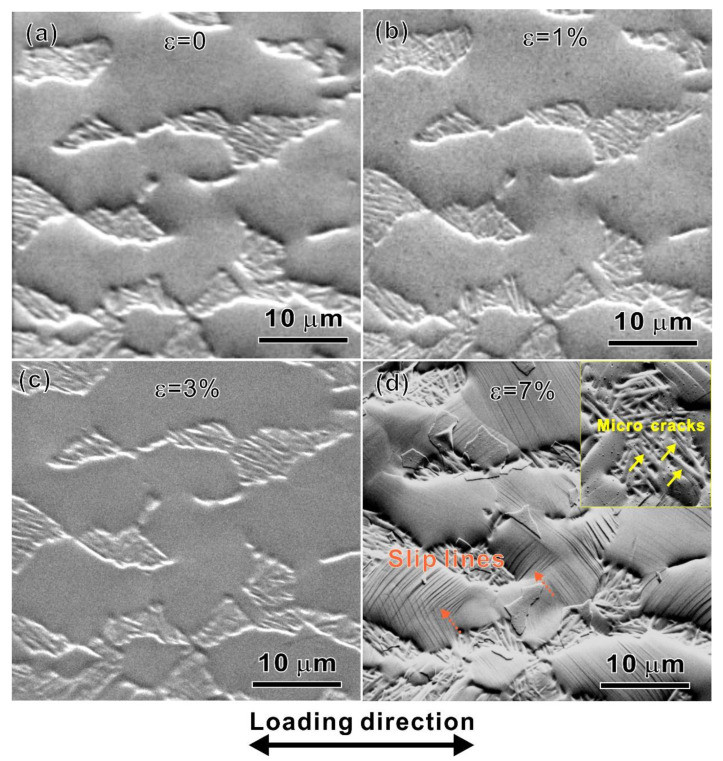
SEM images at the observation area with strains of (**a**) 0%; (**b**) 1%; (**c**) 3%; and (**d**) 7%, respectively.

Figure 8 presents EBSD IPF maps of the observation area corresponding to the strains of 0%, 1%, 3%, and 7%, respectively, during the quasi-in situ tensile test. The tensile strain of 1% is close to the yield strain, and the strain of 7% is close to the necking strain. The LD is indicated by an arrow. The quality of the IPF maps decreases with the increase in strain. Nevertheless, as the strain reaches 7%, part of the EBSD results can still be analyzed. The local misorientation derived directly from the EBSD orientation data was calculated by the KAM method [34,35]. A relation between the misorientation angle (*θ*) and the GND density (*ρ_GND_*) can be expressed by [36,37]

(4) $$\rho_{GND}=\frac{2\theta }{lb}$$

where *l* is the unit length and *b* is Burger’s vector. *b* = $\frac{a}{3}11\bar{2}0$ since the 〈a〉 type slip is an easy mode in an HCP-structured crystal. Figure 9 is a map of GND density of the specimens from *ε* = 0% to 7%. For the specimen at *ε* = 0%, the distribution of the GND density is not homogeneous within the grains and is elevated with increasing tensile strain, as shown in Figure 9a–d. Figure 10a–d shows the SEM observations of surface morphology evolution on the fixed site under different applied strains. No slip lines or slip steps appeared though the applied strain exceeded the yield, as shown in Figure 10a–c. It can be found that the grains’ colors in the four IPF figures have not changed much compared with those of the original state, indicating that the orientations of the grains changed a little even if the slip lines have appeared under the strain of 7%, as shown by the dotted arrows in Figure 10d. Thus, the dislocation movement within grains and deformation compatibility are the main contributions to the deformation evolution. To reveal the deformation evolution behavior, the dislocation density in an *α_p_* grain adjacent to the *α_s_* grain was calculated under different strains, as indicated by the white solid circle in Figure 8a–d and the red dotted circle in Figure 9a–d. The dislocation density of the *α_p_* grain increases from 13.5 × 10^14^ m^−2^ at *ε* = 0% to 22.7 × 10^14^ m^−2^ at *ε* = 7%. Unfortunately, the dislocation density in the lamellar *α_s_* laths could not be calculated because the deformed fine *α_s_* laths are too thin to be detected. However, the SEM observation at *ε* = 7% indicates that there are a lot of micro-cracks between the *α_s_* laths, as shown by the solid arrows in the inset in Figure 10d, indicating the poor strain hardening ability of the lamellar *α_s_*. Therefore, the *α_p_* grains undertake the main deformation.

The quasi-in situ test makes up for the weakness that the influence of a bimodal microstructure on the deformation process is not detected in the DIC experiment. The DIC measurement can only explain the activity in macro zones during deformation, while the quasi-in situ experiment indicates that there is deformation compatibility between the “soft” *α_p_* grains and “hard” lamellar *α_s_* at the micro-scale. The combination of the two experimental methods would make the understanding of the whole deformation process more profound.

## 5. Conclusions

The in situ DIC characterization of the tensile deformation behavior of the bimodal Ti-6Al-4V alloy was conducted. The findings show that several discrete local plastic deformation areas in the bimodal Ti-6Al-4V specimen had already appeared in the elastic deformation stage and extended to connect to each other when yielding is about to occur. After yielding, the scale of the strain accommodation region is about three times that of the strain concentration region. Such a stress concentration cannot be alleviated until the dislocations generated can supplement the strain gradient caused by the strain concentration. The quasi-in situ experiment reveals that the *α_p_* grains undertake the main deformation and there is deformation compatibility between the “soft” *α_p_* grains and “hard” lamellar *α_s_* at the micro-scale.

## Data Availability

The data presented in this study are available on request from the corresponding author.
